# Chronic Alcohol Exposure Alters Behavioral and Synaptic Plasticity of the Rodent Prefrontal Cortex

**DOI:** 10.1371/journal.pone.0037541

**Published:** 2012-05-30

**Authors:** Sven Kroener, Patrick J. Mulholland, Natasha N. New, Justin T. Gass, Howard C. Becker, L. Judson Chandler

**Affiliations:** 1 School of Behavioral and Brain Sciences, The University of Texas at Dallas, Richardson, Texas, United States of America; 2 Department of Neurosciences, Medical University of South Carolina, Charleston, South Carolina, United States of America; 3 Department of Psychiatry and Behavioral Sciences, Medical University of South Carolina, Charleston, South Carolina, United States of America; Institut National de la Santé et de la Recherche Médicale, France

## Abstract

In the present study, we used a mouse model of chronic intermittent ethanol (CIE) exposure to examine how CIE alters the plasticity of the medial prefrontal cortex (mPFC). In acute slices obtained either immediately or 1-week after the last episode of alcohol exposure, voltage-clamp recording of excitatory post-synaptic currents (EPSCs) in mPFC layer V pyramidal neurons revealed that CIE exposure resulted in an increase in the NMDA/AMPA current ratio. This increase appeared to result from a selective increase in the NMDA component of the EPSC. Consistent with this, Western blot analysis of the postsynaptic density fraction showed that while there was no change in expression of the AMPA GluR1 subunit, NMDA NR1 and NRB subunits were significantly increased in CIE exposed mice when examined immediately after the last episode of alcohol exposure. Unexpectedly, this increase in NR1 and NR2B was no longer observed after 1-week of withdrawal in spite of a persistent increase in synaptic NMDA currents. Analysis of spines on the basal dendrites of layer V neurons revealed that while the total density of spines was not altered, there was a selective increase in the density of mushroom-type spines following CIE exposure. Examination of NMDA-receptor mediated spike-timing-dependent plasticity (STDP) showed that CIE exposure was associated with altered expression of long-term potentiation (LTP). Lastly, behavioral studies using an attentional set-shifting task that depends upon the mPFC for optimal performance revealed deficits in cognitive flexibility in CIE exposed mice when tested up to 1-week after the last episode of alcohol exposure. Taken together, these observations are consistent with those in human alcoholics showing protracted deficits in executive function, and suggest these deficits may be associated with alterations in synaptic plasticity in the mPFC.

## Introduction

The prefrontal cortex (PFC) is involved in executive cognitive processes that include supervisory control over impulsive behaviors and the ability to flexibly shift attentional processes as the situation demands. Imaging studies in humans show functional changes in the PFC of both abstinent and non-abstinent alcoholics, including changes in response to cues associated with drinking [1], [2]. Chronic alcohol exposure is also associated with executive dysfunction and with changes in grey and white matter volume in the PFC [3]. Thus, chronic ethanol exposure may induce changes in the PFC that are associated with cognitive impairments, impulsivity and maladaptive decision-making. A confound of these observations in humans is that deficits could reflect a pre-existing phenotype and may not be a direct result of alcohol exposure. Therefore, controlled studies in animal models are needed to better understand how excessive and repeated episodes of alcohol exposure alter PFC function and behavioral control. NMDA receptor (NMDAR)-mediated glutamatergic neurotransmission is required for several forms of neuronal plasticity. Alterations in glutamate neurotransmission in prefrontal-limbic circuits have been implicated in the development of addiction to psychostimulants [4] and may play a similar role in the development of alcohol dependence. Acute alcohol exposure inhibits NMDARs at pharmacological concentrations associated with intoxication [5], [6]. In contrast, chronic alcohol exposure has been reported to increase the synaptic expression of NR2B subunit-containing NMDARs [7]–[13]. This increase presumably occurs as a homeostatic adaptive response to the prolonged reduction of NMDAR activity in the presence of alcohol. NMDARs containing the NR2B subunit have been especially implicated in synaptic plasticity and alterations in learning and memory [14], [15]. Using a mouse model of alcohol dependence that involves repeated cycles of alcohol exposure, the goal of the present study was to determine whether the plasticity of medial PFC (mPFC) is altered in response to chronic alcohol exposure. Specifically, we hypothesized that CIE exposure increases the synaptic expression of NR2B receptors in mPFC pyramidal neurons that in turn promotes persistent alterations in synaptic plasticity. In acute brain slices from adult animals, we found that CIE exposure resulted in an increase in the NMDA/AMPA current ratio that was still present 1-week after the last episode of ethanol exposure. Consistent with a selective increase in synaptic NMDA currents, Western blot analysis of the insoluble PSD containing membrane fraction revealed increases in NR1 and NR2B subunits but no change in GluR1 subunits in tissue examined immediately after the last episode of alcohol exposure. However, the increase in NR1 and NR2B was no longer present when examined 1-week after the last episode of ethanol exposure. At the structural level, analysis of dendritic spines revealed a selective increase in the density of mature (mushroom shaped) spines that persisted throughout 1 week of withdrawal. CIE exposure and withdrawal was also associated with aberrant expression of NMDAR-mediated STDP. Lastly, behavioral studies using a mPFC-dependent task showed that CIE exposure was associated with deficits in behavioral flexibility that persisted up to 1-week after the last period of ethanol exposure. These results indicate that chronic ethanol exposure induces changes in PFC plasticity, which may contribute to a loss of appropriate attentional control over behavior.

## Results

### CIE-treatment alters the ratio of NMDA and AMPA receptor currents

**Figure 1:**
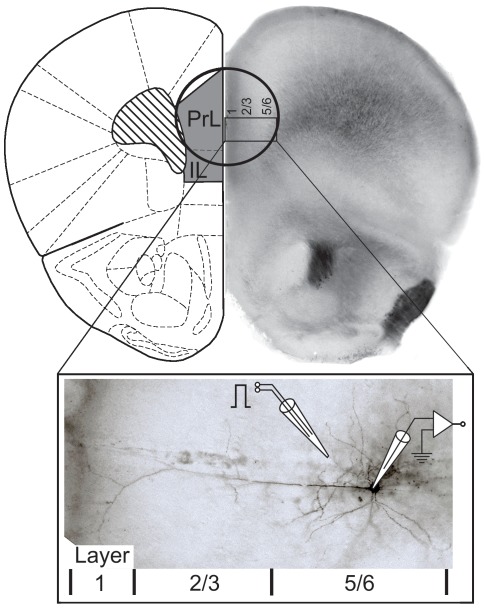
Schematic of the prefrontal cortex (PFC) demonstrating location of tissue punches and neurons used for electrophysiology experiments and morphological analysis. *Top:* Tissue punches used for western blot analysis centered on the prelimbic (PrL) PFC. Shown are sections of the mouse brain at Bregma 2.00 mm. *Bottom*: The box shows a bright-field image of a biocytin-labeled layer V pyramidal neuron in the PrL PFC. The inserts show a typical arrangement of the recording and theta-glass stimulation electrode, which was placed within the same layer as the recorded neuron near the basal dendrites.

**Figure 2:**
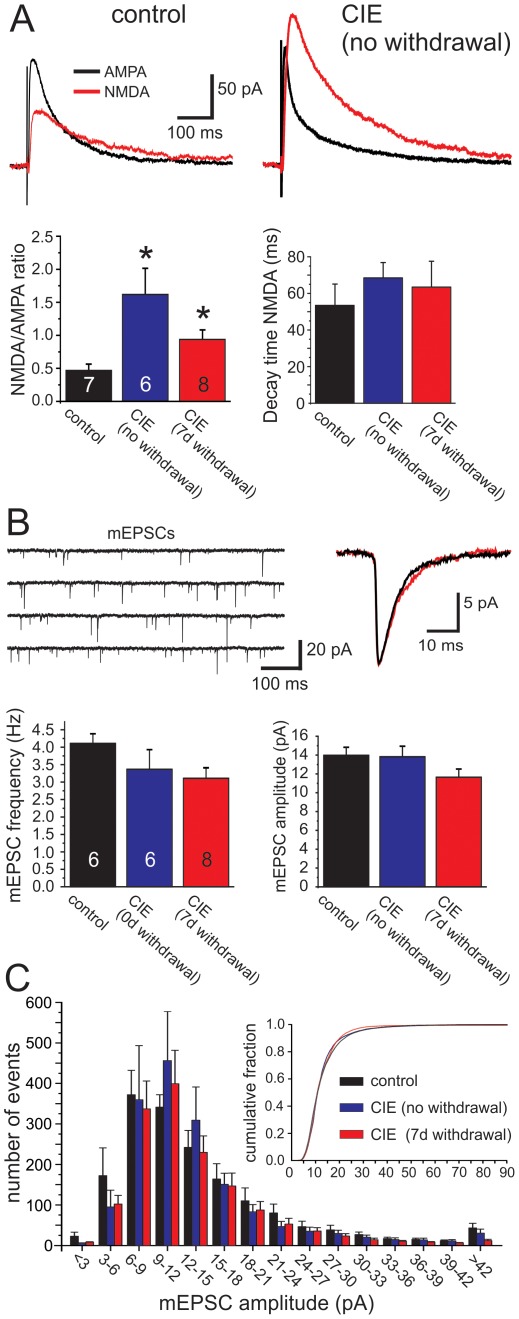
Chronic intermittent ethanol exposure (CIE) increases the ratio of NMDA to AMPA currents independent of alterations in synaptic glutamate release. A) Examples of EPSCs from neurons of a control and CIE-exposed mouse. Left: The NMDA/AMPA ratio was significantly larger in slices from control versus CIE exposed mice. Right: Changes in the amplitude of the NMDAR current was not accompanied by increases in the decay time constant. B) CIE exposure did not affect the frequency or average amplitude of mEPSCs. The top left shows representative traces of pharmacologically isolated mEPSCs in a slice from a control mouse. The right insert shows averaged mEPSCs from a control (black) and CIE (no-withdrawal) mouse, respectively. *p<0.5 significantly different from the vehicle control group (ANOVA and post-hoc analysis using unpaired Student's t-tests). C) Histogram of the amplitude distribution of mEPSCs from all cells over 10 min of recording (control, black; CIE no-withdrawal, blue; CIE 7 days withdrawal, red). The insert shows the same data replotted as cumulative frequency distribution to show that the relative amplitude distribution of synaptic events did also not change.

Because NMDA and AMPA receptors are known targets of alcohol and are critical mediators of synaptic plasticity, we investigated CIE-induced changes in glutamatergic neurotransmission in the mPFC (Figure 1). In the first set of experiments, we used patch-clamp electrophysiology in acute brain slices obtained from control and CIE exposed mice and measured the relative contribution of NMDA and AMPA currents to the total excitatory synaptic transmission of layer V pyramidal neurons. As shown in Figure 2A, CIE exposure increased the ratio of synaptically evoked NMDA- to AMPA receptor-mediated currents. This increase was observed in slices from mice obtained either immediately or 1-week after the last episode of alcohol exposure, indicating this was a persistent change in glutamatergic neurotransmission. Changes in evoked synaptic currents were also found to be independent of changes in action potential-independent mEPSCs (Figure 2B). Neither the frequency nor the average amplitude of mEPSCs differed between slices from control and CIE exposed mice, indicating that CIE exposure did not affect presynaptic glutamate release. Therefore, the observed changes in NMDA/AMPA ratio can most likely be attributed to enhanced postsynaptic NMDAR activity.

### CIE-treatment alters the expression of NR1 and NR2B NMDA receptor subunits and the morphology of spines in medial PFC

**Figure 3:**
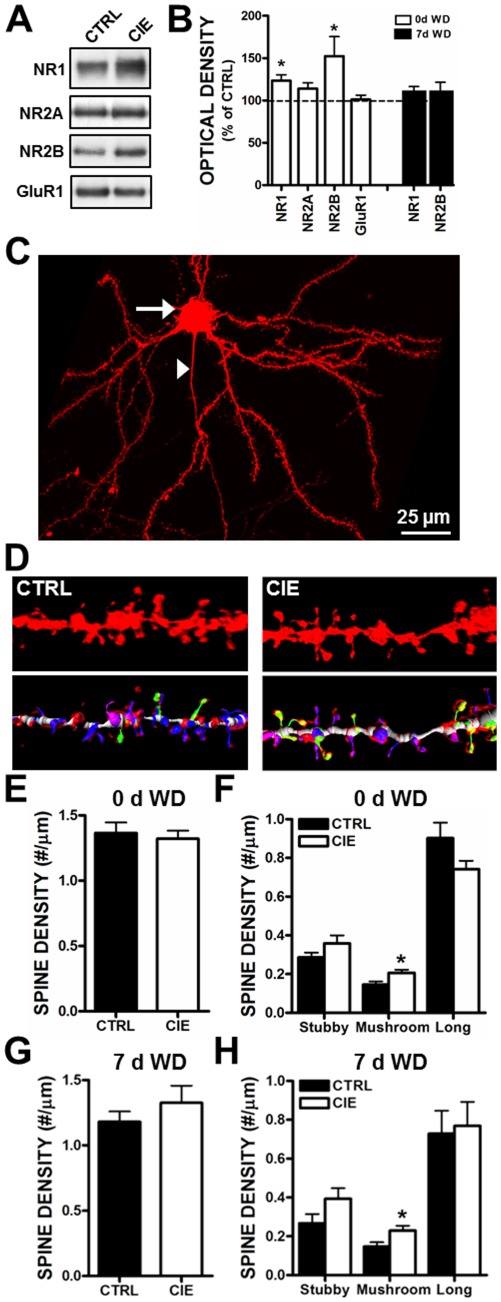
Chronic intermittent ethanol (CIE) increases the density of mature dendritic spines in layer V mPFC pyramidal neurons and produces a transient increase in NMDARs. A) Representative blots of NMDAR and AMPAR subunits in control and CIE exposed mice. B) CIE significantly increased expression of NR1 and NR2B subunits of NMDARs in a PSD-enriched fraction (n = 6–8 mice/group; two-tailed t-tests; *p<0.05) in tissue obtained immediately after the last episode of alcohol exposure. The levels of NR1 and NR2B had returned to baseline after 1- week of withdrawal (WD). C) Representative image of the basal dendrites and dendritic spines of a layer V pyramidal neuron in the mPFC (arrow shows cell body and arrowhead denotes axon). D) Representative images of diolistic labeling of basal dendrites from control and CIE exposed mice. Also shown is automated filament detection and classification of the dendritic shaft (grey) and spines (n = 5–6 mice/group; stubby = red, long = blue, mushroom = green, filopodia = pink). E, G) Total spine density was not altered between control and CIE exposed mice at 0 d or 7 d WD (SAS Proc Mixed model; p>0.05). F, H) The density of mushroom spines was increased by CIE exposure at both time point (SAS Proc Mixed model; *p<0.05).

To determine the effect of CIE exposure on the synaptic expression of NMDARs, we prepared a PSD-enriched fraction from tissue punches taken from mPFC (Figure 1) and carried out immunoblot analysis of NMDAR and AMPAR subunits. Consistent with previous studies in other brain regions and neuronal preparations [7], [11], [16]–[21], we found a significant increase in NR1 and NR2B subunit expression in the mPFC following CIE exposure. In contrast, the expression of NR2A and GluR1 subunits did not significantly differ between the control and CIE exposure groups (Figure 3A, B). These observations are consistent with the selective increase in the NMDA currents (Figure 2) measured immediately after the last episode of alcohol exposure. However, in tissue punches obtained from mice that had undergone 1-week of withdrawal from alcohol exposure, the levels of NR1 and NR2B in the insoluble PSD fraction had returned to baseline. This was unexpected in light of the continued increase in the NMDA/AMPA current ratio at this same withdrawal time-point (Figure 2). At the structural level, we have previously shown that chronic ethanol exposure of primary hippocampal neuronal cultures results in increases in the size of dendritic spines [22]. These in-vitro observations are supported by in-vivo chronic ethanol studies of changes in the density and/or morphology of dendritic spines [23]–[25]. Using diolistic labeling procedures coupled with 3D image analysis, we compared CIE-induced neuroadaptations of dendritic spine density and morphology of basal dendrites of layer V mPFC pyramidal neurons (Figure 3C, D). CIE exposure did not alter the total spine density measured either immediately after the last episode of alcohol exposure (Figure 3E) or following 1-week of withdrawal (Figure 3G). However, there was a small but significant increase in the density of mature, mushroom-type spines, which appeared to be off-set by a reduction in the density of long spines (Figure 3F). Dendrite diameter and volume were not altered by CIE, nor were spine length, diameter, or neck volume at either time points (data not shown). CIE significantly reduced long spine volume 7 days after ethanol exposure (F(1,8) = 5.5; *p*<0.5; control, 0.203±0.01; CIE, 0.153±0.02, not shown).

### Effects of CIE-treatment on STDP in the medial PFC

**Figure 4:**
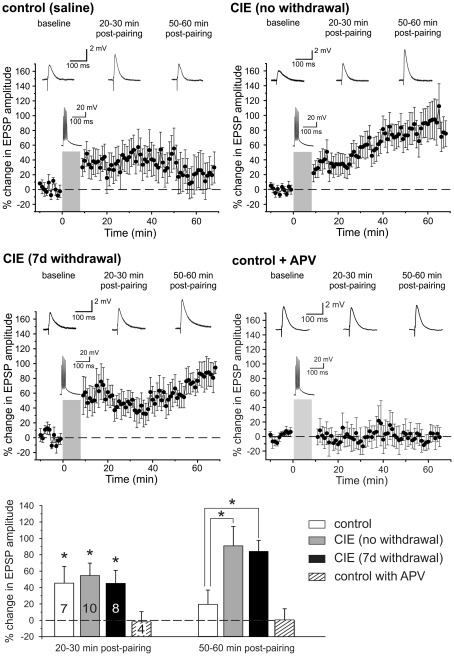
Chronic intermittent ethanol (CIE) exposure alters the time course of STDP. EPSPs were induced by focal stimulation close to basal dendrites of the recorded neuron in layers V/VI. Pairing an EPSP with a burst of postsynaptic action potentials lead to consistent increases in all 3 treatment groups (control, CIE no withdrawal, and CIE followed by 7 days of withdrawal) at 20–30 minutes post pairing. However, at 50–60 minutes post-pairing the EPSP amplitude in CIE exposed mice was significantly increased relative to controls in both the no-withdrawal and one-week withdrawal group. Induction of STDP was NMDAR-dependent: In the presence of the NMDAR antagonist APV the burst of postsynaptic action potentials did not result in a significant change from baseline in slices prepared from control animals. The bar graph shows the relative increase in EPSP amplitude relative to the 10 minutes baseline at 2 different time points (20–30 min and 50–60 min post STDP induction, respectively) for the 4 groups. At 20–30 min, the EPSP amplitude was significantly increased over baseline in all groups. The relative amount of LTP at 50–60 minutes post-pairing was significantly different between control and CIE mice. This was due both to a decrease in potentiation at the late time-point and to the continued increase in LTP in both CIE-exposed groups of mice. *p<0.5 significantly different from baseline (20–30 min) or the vehicle control group (50–60 min), respectively (Repeated measures ANOVA and post-hoc analysis using unpaired Student's t-tests).

To determine whether CIE exposure also alters plasticity of glutamatergic synapses in the mPFC, we compared STDP in slices from control mice and from CIE exposed mice that were sacrificed either immediately or 1-week after the last episode of alcohol exposure. When EPSPs were paired with a postsynaptic burst of action potentials, the amplitude of the synaptic response increased in all three experimental groups (Figure 4). Analysis of the EPSP amplitude revealed a significant main effect of time (F(2,68) = 5.86; *p*<0.0001)), and an interaction between the treatment groups and time (F(2,136) = 1.36; *p* = 0.0088)). Post-hoc comparisons showed that while the amount of potentiation of the EPSP 20–30 min after STDP-induction did not differ between control and CIE exposed mice, the amplitude of the EPSP continued to increase over the entire duration of the recording period in the CIE exposed groups (Figure 4). This was in contrast to STDP in control mice in which the amplitude of the EPSP either reached a stable plateau or even slightly decreased towards baseline levels in some cells. Bath application of APV prevented the increase in EPSP amplitude, indicating that induction of STDP was NMDAR-dependent (n = 4; Figure 4). Thus, these studies demonstrate that CIE exposure resulted in persistent alterations of NMDAR-mediated STDP in deep-layer neurons of the mPFC. The above studies indicate that CIE exposure results in functional and structural changes of glutamatergic synapses of deep-layer neurons in the mPFC.

### The effects of CIE-treatment on cognitive flexibility

**Figure 5:**
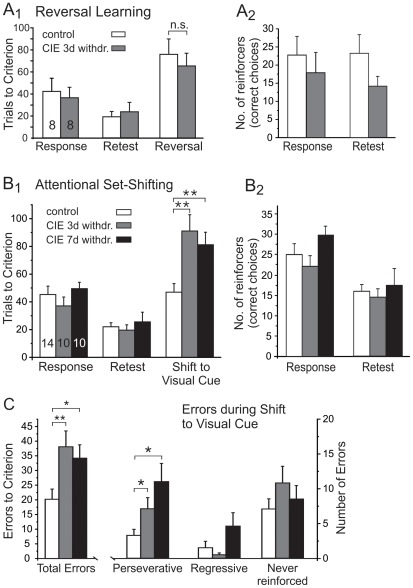
Chronic intermittent ethanol (CIE) exposure reduces the ability of mice to shift attention towards previously unrewarded stimuli in order to learn a new response strategy. A) CIE does not interfere with the ability to reverse a response discrimination in a T-maze. Mice were trained on a response discrimination (Response) that required them to always turn towards one side to obtain food reward. After 3 cycles of CIE or air exposure and 3 days of withdrawal, mice were retested (Retest) using the same turn discrimination. Animals in both groups showed a small improvement in performance on Retest day, indicating that CIE did not impair the retention of the previously learned strategy. On the following day, mice were required to reverse their strategy (Reversal) and turn towards the opposite arm to obtain the reward. The bar graph in A1 shows the total number of trials to criterion on the 3 test days for mice in the control (n = 8) and CIE (n = 8) groups. Data in A2 shows the number of reinforcers earned (i.e. the number of correct choices) during training (Response and Retest Day). No differences were observed between the treatment groups on any test day. B, C) Attentional Set-shifting is impaired in CIE exposed mice. Mice were trained on a response discrimination task as described in *A* with the addition of a visual cue (Response). After 3 cycles of CIE or air exposure and 3 or 7 days of withdrawal, mice were retested on the Response Discrimination (Retest). On the following day, mice were trained to shift attention to the visual cue to obtain food reward (Shift to Visual Cue). B1) CIE exposed mice required significantly more trials to reach performance criterion on Reversal Day. B2) Changes on Reversal Day were not due to differences in association strength established during training. The number of reinforcers earned (i.e. the number of correct choices) was not different in all treatment groups. C) Analysis of the types of errors committed on Reversal Day revealed that CIE exposed mice made significantly more perseverative errors than mice in the control group. Other types of errors did not differ significantly, indicating that once the new strategy was acquired, the mice had no difficulty in maintaining the new response strategy. All data are expressed as means ± SEM. **p<0.01 and *p<0.05 significantly different from the vehicle control group (ANOVA and post-hoc analysis using unpaired Student's t-tests).

The ability to flexibly direct cognitive resources as the situation demands is a common index of executive functioning, and alterations in mPFC function are associated with deficits in attentional control of behavior. We therefore utilized a maze-based procedure to measure both Reversal Learning and Attentional Set-shifting. To assess the effects of CIE exposure on Reversal Learning, the performance of mice on day 1 (Response Discrimination) was used to separate them into Control (n = 8) and CIE exposure (n = 8) groups to equally balance performance levels before the start of the CIE exposure procedure. Accordingly, both groups took an equivalent number of training trials to achieve criterion on day 1 of training (Figure 5A), and there were no differences in the number of probe trials required to complete training (control, 1.25±0.16; CIE, 1.0±0.0; not shown). After completion of response-discrimination training, mice were subjected to either air or the CIE exposure procedure. Three days after the last episode of alcohol exposure (and a total of 24 days after the initial response-discrimination training), the mice were re-tested on the response-discrimination task. As shown in Figure 5A, in both the control and CIE exposed groups the average number of trials to completion on the Retest day was similar to the number on the Response day, indicating that neither CIE nor air exposure altered the mouse's ability to remember the previously learned reward association. Similarly, the average number of reinforcers, i.e. correct responses that resulted in food reward, was also not different between the control or CIE exposed groups on either training days (Response and Retest) (Figure 5B). Thus, on average, the learning history of the animals in all groups was comparable. On the following day (Reversal Day), mice were tested for their ability to reverse their behavioral response and turn towards the arm opposite of the one that was baited on the previous day. As shown in Figure 5A, CIE exposure did not impair the ability of mice to successfully reverse their strategy in this task. Animals needed a comparable amount of trials to criterion, and the number of probe trials to reach this criterion did not differ between groups (control, 1.25±0.25; CIE, 1.125±0.125, not shown). These observations indicated that CIE exposure did not alter the ability of the mice to perform a simple reverse-discrimination procedure. In a separate group of animals, we next determined whether CIE exposure affected the animals' ability to perform a more complex Attentional Set-shifting procedure. The initial response discrimination portion of this task was similar to the Reversal Learning task. However, in the Attentional Set-shifting task, a visual cue is also present whose location varies pseudorandomly, and which has to be ignored by the mouse during the response discrimination training phase. As shown in Figure 5B, there were no differences in the number of training trials for the control and CIE exposed groups to achieve criterion on day 1 of training. Similarly, the number of probe trials required also did not differ between the groups (control 1.5±0.29; CIE 3 days withdrawal, 1.25±0.25; CIE 7 days withdrawal, 1.5±0.25). Following CIE exposure and either 3 or 7 days of withdrawal, mice were retested on the response discrimination phase of the task. Animals in all 3 treatment groups required fewer trials to criterion on the Retest day compared with the performance on the initial Response Discrimination day (Figure 5B), indicating that animals remembered the reward association over 24–28 days, and that CIE did not negatively impact their ability to recall this information. As was true for the simple Reversal Learning task described above, the average number of reinforcers, i.e. correct responses that resulted in food reward, was also not different between the control or CIE exposed groups on either training days (Response and Retest) (Figure 5). Thus, on average, the learning history of the animals in all groups was comparable. On the next day, mice were trained to employ a new strategy that required them to attend to the location of the visual cue (set-shift). As shown in Figure 5B, CIE exposed mice were significantly impaired in their ability to employ the new strategy to obtain food pellets. Analysis of the number of trials to reach the criterion on Shift to Visual-Cue Learning day revealed a significant main effect of treatment (F(2,62) = 13.1, *p*<0.0001), and a significant effect of choice type (F(1,62) = 8.66, *p* = 0.005). There was no significant treatment×choice type interaction (F(2,62) = 0.459, NS), indicating that all mice in all groups made more correct than incorrect choices over the course of training. The total number of trials to reach criterion and the number of errors made were both significantly higher in CIE mice when compared to controls (Figure 5B, C). The number of probe trials to reach this criterion did not differ between groups (control 1.14±0.1; CIE 3 days withdrawal, 1.4±0.25; CIE 7 days withdrawal, 1.6±0.25). A separate analysis conducted on the errors committed during the set-shift revealed a significant main effect of treatment (F(2,93) = 5.02, p = 0.009), a significant effect of error type (F(2,93) = 13.35, *p*<001), and a significant treatment×error type interaction (F(4,93) = 2.47, *p* = 0.049). Post-hoc comparisons showed that CIE-treated mice committed significantly more perseverative errors, but not more regressive or never-reinforced errors during the set-shift (Figure 5C). Thus, CIE exposure reduced the cognitive flexibility of the mice and altered their behavioral response to changing environmental demands.

## Discussion

The goal of the present study was to determine whether chronic alcohol exposure altered the plasticity of the mPFC. Using a mouse model in which chronic alcohol dependence is induced by repeated cycles of exposure to alcohol vapors [26]–[29], we observed that chronic alcohol exposure lead to persistent increases in NMDA/AMPA current ratio at glutamatergic synapses of layer V pyramidal neurons in mPFC slices. This increase was due to a selective enhancement of NMDA currents that persisted for at least 1 week of withdrawal. We also observed an increase in expression of NR1 and NR2B subunits of the NMDA receptor in the insoluble PSD fraction, but this increase was more transient and was no longer observed after 1 week of withdrawal. In contrast to changes in NMDA receptors and currents, CIE exposure did not alter AMPA currents or expression of AMPA GluR1 subunits. CIE exposure also did not alter presynaptic glutamate release as there was no change in mEPSC frequency. We also used diolistic labeling procedures to examine dendritic spines and found that while chronic ethanol did not alter the overall density of spines on basal dendrites of deep-layer pyramidal neurons, there was a significant increase in the number of mature spines that was still present 1 week after withdrawal from the last vapor inhalation exposure. As a direct measure of synaptic plasticity, we examined STDP in the acute slice preparation and found that CIE exposure was associated with an aberrant form of enhanced NMDAR-mediated plasticity. Finally, we used a PFC-dependent task that assesses different components of executive function and found that while CIE exposure did not alter acquisition or recall of a previously learned strategy, it did attenuate the ability of mice to adjust their strategy in response to changing task rules. Taken together, these results indicate that CIE exposure alters structural, functional and behavioral plasticity of the mPFC. Previous patch-clamp slice electrophysiology studies have shown that acute ethanol inhibits NMDA (but not GABAA) currents in the mPFC and reduces NMDA-dependent Up-states that underlie persistent activity in this brain region [30], [31]. Following chronic exposure, NMDARs adapt to the inhibitory effects of alcohol by increasing their excitatory activity via enhanced expression at the synapse [32], [33]. In the present study, we observed similar changes in the mPFC of adult mice. Thus, the redistribution of NMDARs to the synapse may sensitize the synapse to subsequent synaptic plasticity events [34]. However, an unexpected observation in the present study was the apparent mismatch in the persistence of CIE-induced increase in synaptic expression of NMDA receptors compared to synaptic NMDA currents. Using Western blotting procedures, we observed a transient elevation of synaptic NR1 and NR2B subunits that returned to baseline levels after 1-week of withdrawal, while electrophysiological measurement of synaptic NMDA currents showed they remained increased (although at a reduced level) after 1-week of withdrawal. The reason for this discrepancy is not clear, but may relate to methodological considerations. For example, while electrophysiological recordings were restricted to layer V pyramidal neurons in the prelimbic subregion of the mPFC, Western blotting was carried out using tissue punches that included not only all layers of the prelimbic cortex, but also portions of the subregions surrounding the prelimbic PFC (e.g. infralimbic ventrally and anterior cingulate dorsally). Layer-specific effects on synaptic transmission have been observed for several drugs of abuse including alcohol [35], [36]. Furthermore, following prolonged CIE exposure, increases in NMDAR number may transition to alterations in NMDA receptor function with a return to baseline density so as to allow for subsequent plasticity events. Alternatively, the different methods may simply differ in their ability to quantify CIE-induced changes in NMDAR expression versus function. Spike timing-dependent plasticity is a well-characterized cellular model of NMDAR-dependent synaptic plasticity that describes changes in synaptic efficacy in response to repeated pairings of near coincident pre- and postsynaptic APs. For the induction of STDP, we used a pattern of high-frequency postsynaptic activation that facilitates induction of Ca^2+^ spikes and NMDAR activation in basal dendrites that leads to LTP of the amplitude of the EPSP [37], [38]. Using this protocol, we observed STDP in mPFC slices from all three treatment groups (i.e. control, CIE-no withdrawal, CIE-1 week withdrawal). However, in CIE exposed mice, induction of NMDAR-dependent STDP was significantly increased compared to that observed in control slices at 50–60 min post-pairing. Consistent with the CIE-induced increase in NMDAR-dependent STDP, it was recently reported that chronic ethanol exposure transforms NMDAR dependent long-term depression (LTD) into LTP in the nucleus accumbens shell [39] and also reduced the threshold required for LTP induction in the hippocampus [40]. Although the exact molecular mechanisms that underlie altered synaptic plasticity that we observed are unclear, they likely involve enhanced Ca^2+^ influx through NR2B-containing receptors. Interestingly, NMDAR on pyramidal neurons of the PFC have distinct biophysical properties compared with those in sensory cortical areas due to a significantly higher NR2B subunit expression in the PFC [41]. Thus, the subunit composition of these NMDAR conveys unique properties to PFC networks that support flexible control of behavior [41], but it may also make the PFC especially vulnerable to the effects of prolonged ethanol [34], [42]. Under conditions that do not favor induction of synaptic plasticity (i.e. passive exposure to alcohol in vapor chambers vs. alcohol self-administration in an operant learning paradigm) AMPA receptors did not appear to be altered by CIE. However, our data clearly do not exclude a role for AMPARs during the induction of NMDAR-dependent synaptic plasticity. As demonstrated in many brain regions, postsynaptic LTP typically requires NMDAR activation and Ca^2+^ influx, which results in the rapid recruitment of AMPA receptors as well as a concomitant modification in the phosphorylation state of synaptic AMPA receptors [43]. Longer (>3 hrs) forms of LTP also require the synthesis of new proteins to sustain a long-term memory [44]. Our physiology experiment only examined short (∼1 hr) changes in synaptic plasticity and thus the role of CIE-induced alterations in NMDAR function (Figures 2 and 3) for long-term synaptic changes is as of yet unclear. However, in addition to an increase in the synaptic targeting of NMDARs, we also found that CIE induced morphological changes in a subpopulation of spines on the basal dendrites of deep-layer pyramidal neurons in the mPFC, consistent with the idea of long-term synaptic plasticity [45]. Specifically, CIE exposure produced a persistent increase in the density of mature, mushroom spines without altering total spine density. This observation is also consistent with findings from a study in the nucleus accumbens that showed similar specific alterations in spine morphology in a model of chronic alcohol drinking and repeated deprivation [23]. Changes in the structural plasticity of dendritic spines have also been observed in response to exposure to psychostimulants [46]. Thus, our data provide further evidence of structural remodeling in response to alcohol and drug exposure.

Although our observations in acute slices demonstrate that CIE exposure alters NMDAR function and NMDAR-dependent synaptic plasticity in the mPFC, it is not clear how these changes will affect neural networks in the mPFC. Based upon previous observations that enhanced expression of NR2B subunits promotes enhanced learning and memory, one might expect that chronic ethanol-induced increases in NR2B receptors in the mPFC would promote enhanced learning and memory. In fact, a recent study using a chronic model of alcohol exposure in rats reported enhanced learning and memory in rats following lower levels of chronic alcohol exposure, but reduced learning and memory at higher levels [47]. However, to our knowledge, there is little evidence of enhanced learning and memory in humans following excessive and repeated episodes of binge-like alcohol exposure. In contrast, we suggest that the CIE-induced alterations in STDP we observed here are an aberrant homeostatic response. This change may lead to aberrant metaplasticity of NMDAR dependent synaptic plasticity [39], [40], [48], which could promote dysfunctional reorganization of prefrontal neuronal networks. The PFC mediates executive functions entailing the coordination, manipulation, and flexible use of information from multiple memory systems [49], [50]. Reversal learning requires flexible switching between cues within a particular stimulus dimension, and it invokes multiple executive functions, including attention, working memory and response inhibition. Perseverative errors serve as an index of how readily animals are able to inhibit the use of the now incorrect strategy and instead attend to previously irrelevant stimuli in order to obtain a goal. Distinct regions of the PFC play a critical role in facilitating different forms of behavioral flexibility. Lesions or blockade of NMDARs in the mPFC has been shown to impair set-shifting and produce a selective increase in perseverative errors, whereas the learning and maintenance of a new strategy remains unaffected, indicating that the mPFC plays a selective role in suppressing the use of a previously relevant but now incorrect strategy [51]–[56]. In contrast, reversal learning is impaired by lesions of the orbitofrontal region of the PFC, which have no effect on set-shifting functions [53], [57]–[59]. Floresco and Magyar [53] have argued that attentional set-shifting is a more complex process than reversal learning. This is because successful attentional set-shifting requires both the suppression of a previously learned strategy and attending to a previously irrelevant stimulus, thus requiring that attention be paid to multiple aspects of the environment [60], [61]. In the present study, we found a dissociation of the effects of CIE on these behavioral measures of cognitive control, suggesting that alcohol exposure disrupts the ability to shift between attentional sets, but does not interfere with hierarchically less complex reversal learning. The CIE-induced deficit in set-shifting was the result of increased perseverative responding and was not due to impairment in the initial acquisition of either a response or visual discrimination. Thus these findings are consistent with the idea that CIE exposure reduces the ability of the mPFC to flexibly modulate behavior during changing environmental situations that require increased attentional control. Medial PFC networks control executive functions and behavioral flexibility, and in humans they may regulate cognitive control over alcohol intake [1], [2]. Thus the CIE-induced changes in glutamatergic transmission in mPFC pyramidal cells that we describe here may contribute to the cognitive impairments and loss of behavioral control seen in alcohol-dependent subjects.

## Materials and Methods

### Ethics statement

All procedures were carried out in accordance with the *NIH Guide for the Care and Use of Laboratory Animals*, and were approved by the I*nstitutional Animal Care and Use Committees of the Medical University of South Carolina* and *The University of Texas at Dallas*, respectively.

### Alcohol dependence model

Male adult C57BL/6 mice (Charles River Laboratories, Kingston, NY) were single-housed under a 12 hr light/dark cycle, with continuous access to food and water. Animals in all groups were a minimum of 70 days old before treatment began. Induction of alcohol dependence followed procedures previously described [62]. In brief, mice were exposed to ethanol vapor (15–17 mg/liter air) or air in inhalation chambers for 16 hrs/day for 4 consecutive days with 8-hr periods of withdrawal separating each exposure. Animals underwent 3 consecutive cycles, each separated by 3 days of withdrawal in their home cages. On the days that animals went into the vapor chambers, ethanol intoxication was initiated by administration of 1.6 g/kg ethanol (8% w/v; IP) together with the alcohol dehydrogenase inhibitor pyrazole (1 mmol/kg). Mice in the control group received equivalent injections of pyrazole in saline (all animals received a total of 12 injections of pyrazole spaced over the 3 week treatment period). Chamber ethanol concentrations were measured daily and blood samples were collected from all mice for blood ethanol analysis to ensure stable BEC (175–225 mg/dl) throughout the exposure period.

### Electrophysiological recordings

Animals were sacrificed for electrophysiological recordings either immediately after the third cycle of CIE or following an additional week of withdrawal. Thus, animals were a minimum of 91 or 98 days old, respectively, at the time of the recordings. Mice were anesthetized with urethane (3 g/kg body weight) and perfused with cold oxygenated (95% O_2_-5% CO_2_) artificial cerebro-spinal fluid (ACSF) consisting of (in mM): 230 sucrose, 1.9 KCl, 1.2 NaH_2_PO_4_, 33 NaHCO_3_, 0.5 CaCl_2_, 6 MgCl_2_, 10 glucose, 0.4 ascorbic acid. Coronal sections of the frontal cortex were cut on a vibratome. Whole-cell current- and voltage-clamp recordings were obtained from pyramidal neurons in layers 5–6 of the mouse infralimbic and prelimbic cortex at 35–37°C using the following recording ACSF (in mM): 126 NaCl, 2.5 KCl, 1.2 Na_2_HPO_4_, 25 Na_2_HCO_3_, 10 glucose, 2 CaCl_2_, and 1 MgCl2. For voltage-clamp recordings, electrodes (2–3 MΩ) were filled with (in mM): 130 CsCl, 20 TEA, 10 HEPES, 2 MgCl_2_, 0.5 EGTA, 4 Na-ATP, 0.3 Na-GTP, 14 phospocreatine, and 2 QX-314. For current-clamp recordings, electrodes were filled with (in mM): 125 K-gluconate, 10 HEPES, 20 KCl, 4 ATP-Mg, 0.3 GTP-Na, 14 phosphocreatine. Recordings were performed using a MultiClamp 700B amplifier (Axon Instruments, Union City, CA). Data acquisition and analysis used Axograph-X software (J. Clements, Sydney, AUS). Access resistance was monitored throughout and a<20% change was deemed acceptable.

### Synaptic stimulation and STDP

We elicited excitatory postsynaptic potentials (EPSPs) and currents (EPSCs) through focal extracellular stimulation close to basal dendrites of the recorded neuron (Figure 1) using theta-glass pipettes (Warner Instruments, Hamden, CT). To study CIE-induced changes in NMDAR- and AMPAR-mediated currents, we calculated the NMDA/AMPA ratio as previously described [63]. In brief, a compound EPSC (consisting of both AMPA and NMDA currents) was first recorded at a holding potential of +40 mV. The AMPA component of the synaptic response was then pharmacologically isolated by bath application of the NMDAR blocker APV (50 µM). This AMPA current was then digitally subtracted from the compound response in the absence of APV to yield the NMDAR current. The peaks of the isolated AMPA and NMDA responses were then divided to yield the NMDA/AMPA ratio and compared across treatment groups. To study changes in STDP, EPSPs were evoked at 0.1 Hz from resting membrane potential until a stable baseline was obtained for at least 10 min. STDP was then induced by 48 pairings of evoked EPSPs and postsynaptic APs at 0.1 Hz. Postsynaptic APs were evoked by brief (2 ms; 2 nA) somatic current injections consisting of 5 pulses (200 Hz). Stimulation of the EPSP preceded the middle AP of the burst by 2 ms. The amount of potentiation or depression of the synaptic response was assessed by calculating the ratio of the average EPSP amplitude 20–30 min and again 50–60 min after STDP induction relative to the average amplitude of the EPSP during the 10 min preceding induction. Changes of the response were assessed using a two-factor analysis of variance, with treatment (control and ethanol) and the two time points (i.e. the no-withdrawal and the 1-week withdrawal condition) as between-subjects factors. Significant main effects were further analyzed with pair-wise comparisons using Student's t-tests. The frequency and amplitude of miniature EPSCs (mEPSCs) were measured using MiniAnalysis (Synaptosoft, Decatur, GA) using a threshold set at two times the RMS baseline noise. The detected events were confirmed as synaptic events by visual inspection. For mEPSCs we collected 9 second long sweeps every 30 seconds for 10 minutes.

### Pharmacological compounds

GABA_A_ receptor mediated currents were blocked by application of picrotoxin (75 µM) to the bath. To block NMDARs we used 50 µM D, L (-)-2-amino-5-phosphonopentanoic acid (APV). Miniature AMPA EPSCs were recorded in the presence of 50 µM APV and 1 µM tetrodotoxin. All reagents were obtained from Sigma (St. Louis, MO).

### Western blot analysis

Triton X-100 insoluble (PSD-enriched) and soluble fractions were prepared from tissue punches of mPFC (Figure 1) immediately following removal of mice from vapor chambers or 1-week post vapor exposure (modified from [64]). In brief, a Dounce homogenate was prepared and centrifuged at 1,000× *g* for 10 min to remove nuclei and debris (P1). The supernatant was spun at 12,000× *g* for 20 min to obtain a P2 fraction. P1 and P2 fractions were resuspended and centrifuged 2× to remove contaminants. The P2 fraction was then resuspended in buffer containing 0.5% Triton X-100 and rotated for 15 min. This fraction was then centrifuged at 12,000× *g* for 20 min to yield soluble and insoluble fractions, and the insoluble fraction was then solubilized into 2% LDS. All fractionation steps were performed at 4°C in the presence of 2 mM Na^+^ pyrophosphate, 1 mM activated Na^+^ orthovanadate, and 1 mM Na^+^ fluoride, containing Complete Protease inhibitor cocktail (Roche Applied Science, Indianapolis, IN). Five µg of protein were separated on 7.5% Bis-TRIS gels, and membranes were then probed for glutamate receptor subunits according to standard immunoblotting procedures. Antibodies used in these studies were GluR1 (1∶1000; Abcam, Cambridge, MA), NR1 (1∶4000; BD Pharmingen, Franklin Lakes, NJ), NR2A (1∶1000; Millipore, Billerica, MA), and NR2B (1∶1000; NeuroMab, Antibodies, Inc. & UC Davis, Davis, CA). Data were analyzed by Student's t-test using GraphPad Prism software.

### Dendritic spine classification and analysis

Dendritic spine labeling followed previously reported methods [65]. One group of mice was sacrificed immediately after the last cycle of vapor chamber exposure, another group was sacrificed following an additional week of withdrawal. Mice were anesthetized and perfused with 0.1 M phosphate buffer followed by 1.5% paraformaldehyde (PFA) in phosphate buffer. Brains were postfixed in 1.5% PFA for 30 min, before coronal sections (150 µm) were prepared on a vibratome. Tungsten particles (1.3 µm diameter) were coated with DiI and delivered diolistically using a Helios Gene Gun (Bio-Rad) fitted with a polycarbonate filter (3.0 µm pore size; BD Biosciences). DiI was allowed to diffuse overnight at 4°C, and sections were postfixed in 4% PFA for 1 h. Confocal images (Leica SP5) of 1st and 2nd order basal dendrites of layer V pyramidal neurons were collected and a filament of the dendritic shaft and spines was created using Imaris XT (Bitplane, Zurich, Switzerland) on deconvolved images. Dendritic spines were classified into 4 categories (long, mushroom, stubby, or filopodia) based on their length and neck and head width, where *L* is spine length, *WH* is spine head width, and *WN* is spine neck width. Very few spines were classified as filopodia (∼1.4% of total) and they were thus excluded from further statistical analyses. Long spines were identified as having a *L*≥0.75 µm and <3 µm, mushroom spines had a *L*<3.5 µm, *WH*>0.35 µm and a *WH*>*WN*, stubby spines had a *L*<0.75 µm, and filopodia were identified as having a *L*≥3 µm. Data from 18 mice (*N* = 4–6/group/time point; 87 dendritic sections; 5737 spines) were analyzed as a mixed model (SAS Proc Mixed) with a first order autoregressive covariance matrix across the sequential slices within mice. Because no difference in spine density was observed between 1st and 2nd order basal dendrites, data were collapsed before subsequent analysis.

### Behavioral testing

Behavioral experiments used methods modified from [54]. In brief, prior to behavioral experiments mice were gradually food-restricted until their weight stabilized at 85% of their free-feeding weight. The general set-up of the maze and the habituation training was identical for the Reversal Learning and the Attentional Set-shifting tasks. Both tasks use a four arm cross maze that converts into a “T” configuration by placing a divider in front of one of the arms. On the first day of habituation, four reward pellets (Cheerios bits) were placed in each of the arms of the maze and mice were allowed to freely explore the maze and to consume the food pellets for 15 min. If a mouse consumed all 16 pellets prior to 15 min, it was removed from the maze and placed in a holding cage while the maze was rebaited, then the mouse was placed back in the center of the maze. On the second day of habituation, arms were only baited with two pellets each (in the middle and at the end of the arms). Again, all arms were rebaited whenever a mouse consumed all 8 food pellets and mice were allowed to explore the maze for a total of 15 min. On the third day of habituation, only one food pellet was placed at the end of each arm. To reach habituation criterion, animals were required to consume all four food pellets on the maze at least 4 times within the 15 min period. All animals in this study reached this criterion on the third habituation day. On the following day the turn bias for the animals was determined. An opaque barrier was placed at the entrance of one of the arms, forming a “T” configuration. In addition, in the Attentional Set-Shifting task a visual cue was placed on the wall of one of the choice arms, close to the entry of the arm (see below). A mouse was placed in the stem arm and allowed to turn left or right to obtain a food pellet. After the mouse chose an arm and consumed the reward, it was returned to the stem arm and allowed to make another choice. If the mouse chose the same arm as on the initial choice, it was returned to the stem arm until it chose the other arm and consumed the food pellet. After choosing both arms, the mouse was placed in the holding cage and the barrier (and the visual cue, if present) was moved to different arms to start a new trial. The initial turn that a mouse made during each trial was counted toward its turn bias, and the direction (right or left) that a mouse turned four or more times over seven trials was considered its turn bias.

#### Reversal Learning task

On the day following turn bias, mice were trained on a response discrimination task (Response discrimination day). For this task, the mice were required to always turn in the opposite direction of its turn bias (left or right) to obtain reward. Mice started from one of 3 arms to discourage them from using an allocentric spatial strategy to locate the food. The order of the start arms alternated in a pseudorandom manner so that the frequency of arms was balanced across blocks of 12 trials. Mice continued training until they reached a criterion of 9 correct choices over 10 consecutive trials. After a mouse achieved this acquisition criterion, a probe trial was administered in which they began the trial from the previously unused fourth arm. If the mice performed the probe trial correctly, then response discrimination training was completed. If a mouse made an incorrect turn, response training was continued until the mouse made five consecutive correct choices, at which time another probe trial was administered. This procedure was continued until the mouse reached the criterion and also made a correct choice on the probe trial. In this study, no mouse required more than 2 probe trials to reach criterion. For each mouse, the total number of trials to reach criterion was recorded. Mice with similar test scores were then pairwise assigned to the CIE and air control group, respectively, to reduce variability between treatment groups based on performance in the Response Discrimination phase of the task. In the afternoon of the Response Discrimination day, mice were then subjected to the first of 3 cycles of ethanol (or air) exposure as outlined above. After the third cycle of CIE or air exposure animals remained in their home cage for 3 days. We chose 3 days of withdrawal to ensure that the performance of animals in the following behavioral tasks was not affected by symptoms of acute withdrawal. Following this withdrawal period, animals were retested on the response discrimination task (Retest day) to assess retention of the previously learned association. Procedures and criterion for the Retest day were identical to those on the initial Response Discrimination day. The following day (Reversal day), animals were trained to reverse their previous association and to turn to the previously unrewarded side in order to obtain food reward. We analyzed the total number of trials to criterion and the number of probe trials.

#### Attentional Set Shifting task

On the day following turn bias (Response Discrimination day), animals were trained to always turn in the opposite direction of their turn bias to obtain reward as described above. This task also used 3 arms as start arms, which were alternated pseudorandomly to balance their frequency across blocks of 12 consecutive trials. However, in the Attentional Set-shifting task, an additional visual clue was present that had to be ignored by the mouse during the training phase. The visual clue consisted of vertical black and white stripes on a thin plastic sheet (13×10 cm) that was attached to the side of one arm opposite the stem arm from which the mouse started the trial. Placement of the visual cue into the right or left arm, respectively, was varied pseudorandomly to balance the frequency of occurrences in each arm across blocks of 12 consecutive trials. Otherwise, training and response criteria on Response Discrimination day were identical to those described for the Reversal Learning task. For each mouse, the total number of trials until criterion was recorded and mice with similar test scores were pairwise assigned to the CIE and air control groups, respectively. In the afternoon of the response discrimination day, mice began the first of three cycles of ethanol (or air) exposure as outlined above. After the third cycle of CIE or air exposure, mice were returned to the animal vivarium for a period of 3 or 7 days until behavioral testing began. As described above for the Reversal Learning task, a 3-day withdrawal period was chosen to minimize effects of acute alcohol withdrawal on behavioral performance. A separate group of animals undergoing 7 days of withdrawal was included to provide a measure of the persistence of the observed effects and to better correlate behavioral performance with electrophysiological measurements. Following the withdrawal period, mice were retested on the response discrimination task (Retest day) to assess retention of the previously learned association. Procedures and criteria for the Retest day were identical to those on the initial Response Discrimination day. On the following day (Shift to Visual-Cue Learning day), mice were then trained to always enter the arm that contained the visual cue, the location of which was again pseudorandomly varied in the left and right arms. Training was continued until a mouse made a correct choice on the probe trial. For each day, we analyzed the total number of trials to criterion and the number of probe trials required to reach criterion. For the Shift to Visual-Cue Learning day, errors were scored as entries into arms that did not contain the visual cue, and they were further broken down into three subcategories to determine whether CIE altered the ability to either shift from the previously learned strategy (perseverative errors) or to maintain the new strategy after perseveration had ceased (regressive or never-reinforced errors) [54]. In order to detect shifts in the strategies that animals used, trials were separated into consecutive blocks of four trials each. A perseverative error occurred when a mouse made the same egocentric response as required during the Response Discrimination day, but which was opposite to the direction of the arm containing the visual cue. Six of every 12 consecutive trials required the mouse to respond in this manner (i.e., enter the arm opposite of the previously learned turn direction). A perseverative error was scored when the mouse entered the incorrect arm on three or more trials per block of 4 trials. Once the mouse made less than three perseverative errors in a block, all subsequent errors were now scored as regressive errors (because at this point the mouse was following an alternative strategy at least half of the time). The third type of error, termed “never reinforced” errors, was scored when a mouse entered the incorrect arm on trials where the visual cue was placed on the same side that the mouse had been trained to enter on the previous day.

#### Data Analysis

The number of trials to criterion obtained from the last day of training (Reversal or Attentional Set-shifting day) for each experiment were analyzed using separate two-way between/within subjects ANOVAs, with drug treatment as the between subjects factor and choice type (correct or error) as the within subjects factor. Data regarding the types of error were analyzed separately using a two-way between/within subjects ANOVA, with drug treatment as the between subjects factor and error type (perseverative, regressive, or never-reinforced) as the within-subjects factor. When a significant main effect of drug treatment was observed, multiple post-hoc comparisons were conducted using Student's t-tests.
